# Supplementary material for: Magneto-optical imaging of thin magnetic films using spins in diamond
Source: Sci Rep. 2016 Mar 14;6:22797. doi: 10.1038/srep22797 (PMC4789603; doi:10.1038/srep22797)
Supplement: Supplementary Information [file srep22797-s1.doc]

**Supplementary Information**

**Magneto-optical imaging of thin magnetic films using spins in diamond**

**David A. Simpson^1,2,*^, Jean-Philippe Tetienne^1,3^, Julia M. McCoey^1^, Kumaravelu Ganesan^1^, Liam T. Hall^1^, Steven Petrou^2,4,5^, Robert E. Scholten^1^, Lloyd C. L. Hollenberg^1,2,3^**

^1^School of Physics, University of Melbourne, Parkville, 3052, Australia

^2^Centre for Neural Engineering, University of Melbourne, Parkville, 3052, Australia

^3^Centre for Quantum Computation and Communication Technology, University of Melbourne, Parkville, 3052, Australia

^4^Florey Neuroscience Institute, University of Melbourne, Parkville, 3052, Australia

^5^Centre for Integrated Brain Function, University of Melbourne, Parkville, Victoria, Australia

*corresponding author [simd@unimelb.edu.au](mailto:simd@unimelb.edu.au)

**Magnetic force microscopy of the magnetic recording media**

Magnetic force microscopy (MFM) is the standard imaging tool used to characterise magnetic recording media. To benchmark our results with this imaging technique we take advantage of the MFM resolution to characterise the size of the magnetic bits from a commercial western digital hard drive with a storage capacity of 1GB. The sample used for MFM imaging was cut from the WD drive approximately 8x8 mm and was mounted on an Asylum Research MFP3D Atomic Force Microscope with a MESP cantilever (Bruker) used for MFM imaging. MFM was performed using a two-pass technique, in the first pass, the tip scans the surface as it would in regular AC mode. In the second pass, referred to as the Nap pass, the tip lifts above the surface by a constant height and images the long-range magnetic forces along that same scan line. The height topography image along with the MFM image of the magnetic recording media are shown in Supplementary Figure 1a and 1b respectively. The acquisition time of each image was 30 minutes for the 40x40 µm area.


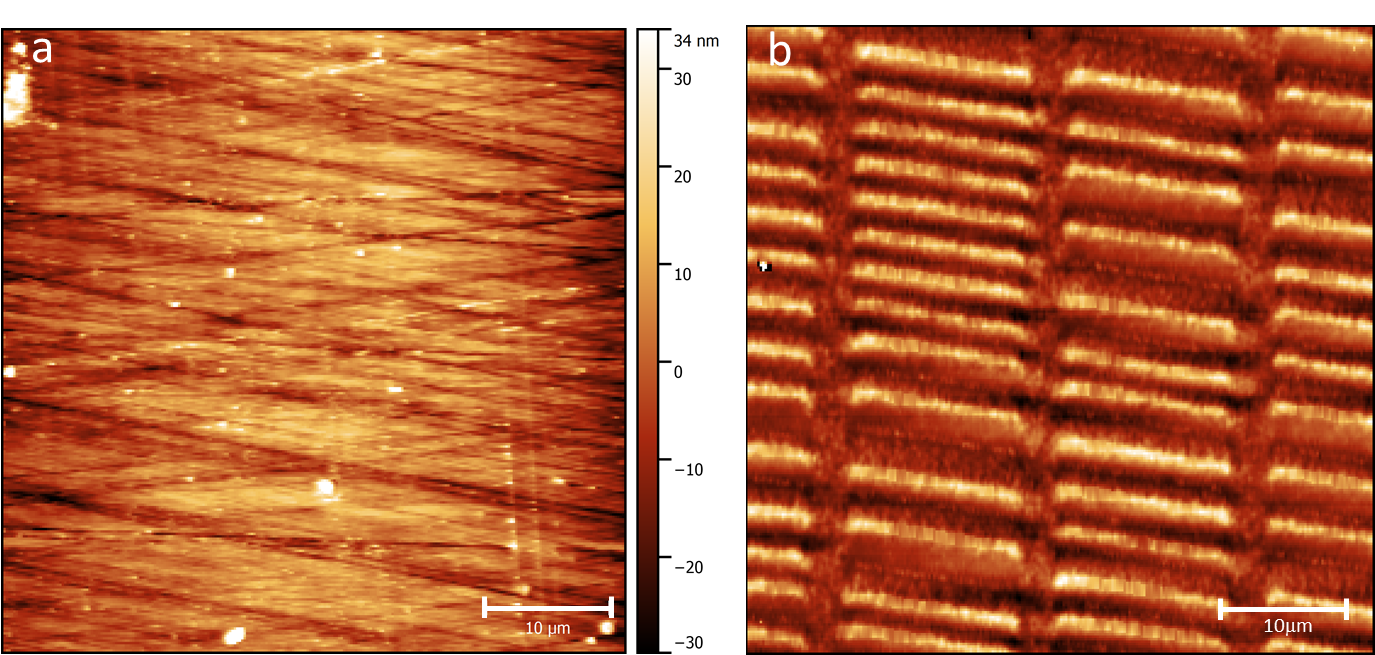


Supplementary Figure 1: AFM and MFM images of the recording media. (a) Height topography of the magnetic drive. (b) Magnetic force gradient image depicting the magnetisation of the magnetic bits.

A line scan perpendicular to the magnetic bits enables the bit spacing to be determined, as shown in Supplementary Figure 3.


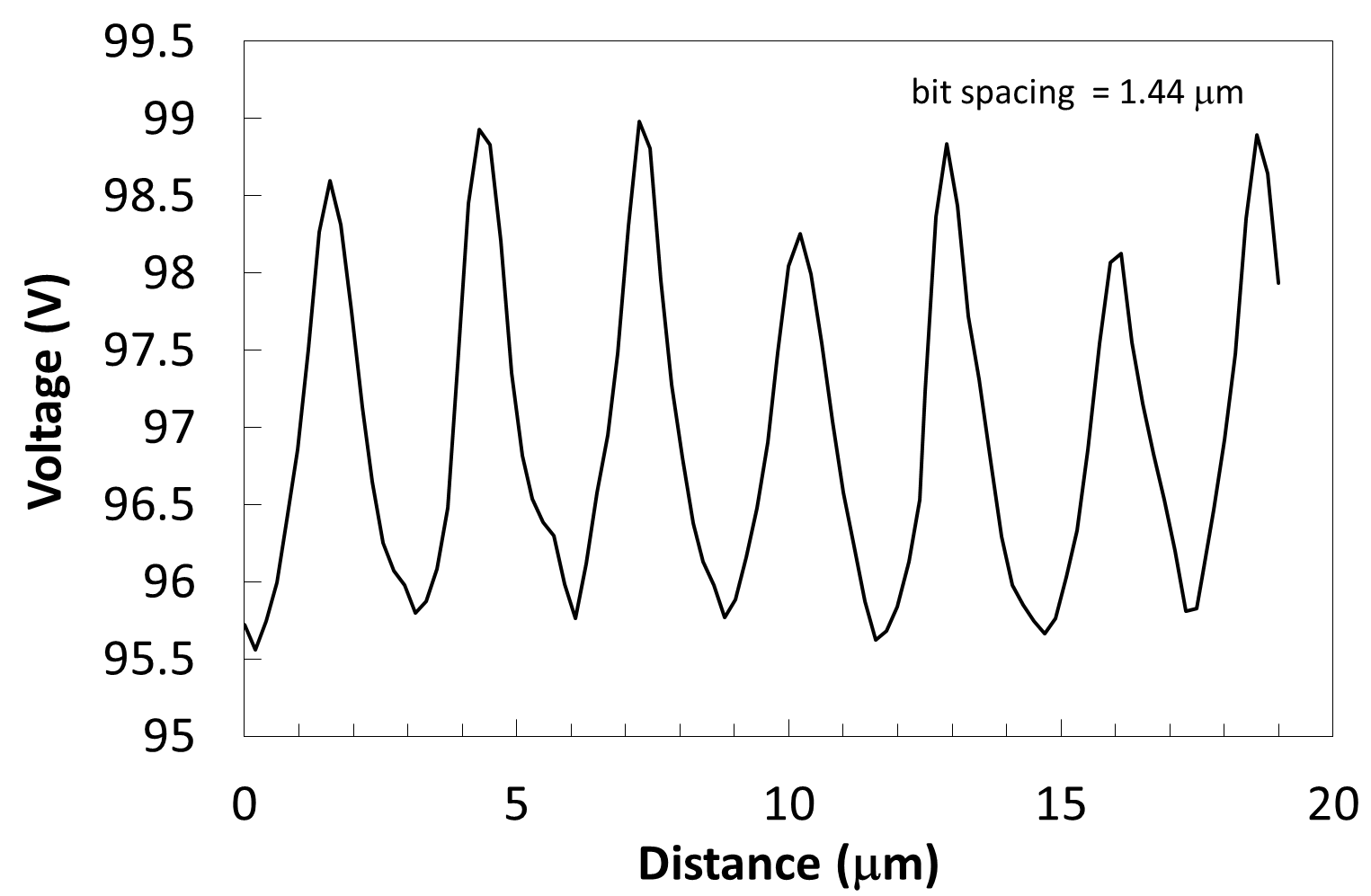


Supplementary Figure 3: Line scan perpendicular to a regular sequence of magnetic bits.

Since the MFM images the magnetic force gradient of the sample, the bit spacing is equal to half the measured period. From the line scan this equates to an average bit spacing of 1.44µm for the regular 010101 magnetisation pattern observed in Supp. Figure 1b. The bit length can be determined directly from the MFM image by taking a line scan parallel the magnetic bits. Supplementary Figure 4 shows the line scan parallel to the bits indicating a bit length of 14.0 ± 0.5 µm with approximately 3µm between the data tracks.


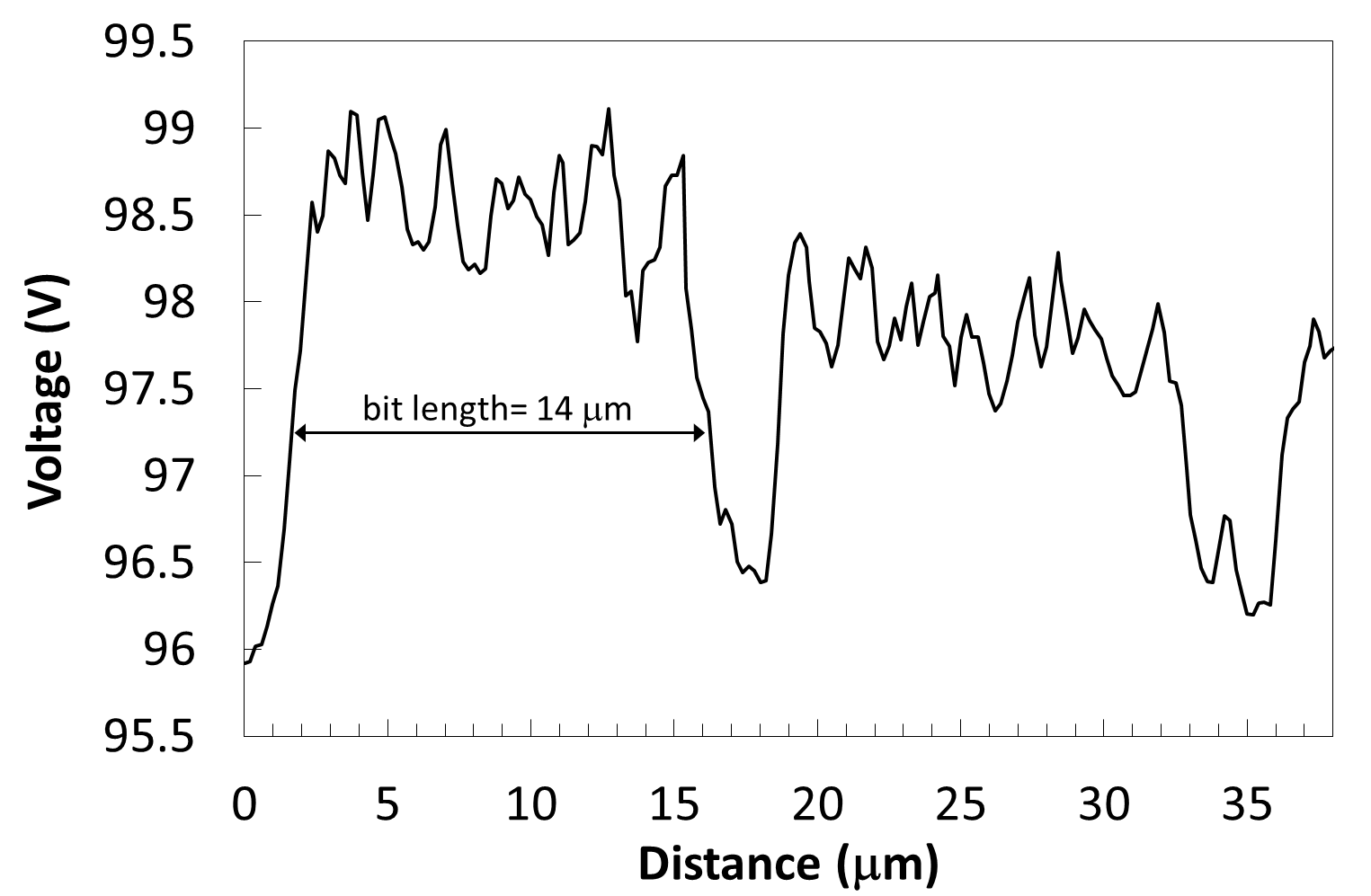


Supplementary Figure 4: Line scan along the magnetic bits. The length of each magnetic bit was 14.0 ± 0.5 µm.

The bit spacing and bit length parameters are used in the micromagnetic model described in the manuscript. Using these parameters the predicted magnetic bit spacing is in excellent agreement with the measured bit spacing (1.47µm) using the magneto-optical diamond microscope as seen in Figure 3b of the manuscript. This verifies the imaging performance of our magneto-optical microscope.

**Spin relaxation imaging**

The spin relaxation time of the diamond imaging chip was measured in the presence of the recording media. Supplementary Figure 5a shows the magnetic image of the drive in the region where the full *T_1_* curves were measured. The two points Sx_1_y_1_ and Sx_2_y_2_ are the points which the full *T_1_* curves are shown in the manuscript (Figure 4b). Supplementary Figure 5b shows the corresponding *T_1_* map for the same area. A stretched exponential is used to fit the full *T_1_* curves at each pixel. The stretched exponential arises from the fact that the NV centres within the imaging chip are located at varying depths from the ion struggle of the N implant. Therefore a distribution of exponential decays is observed . We do not see any correlation with the *T_1_* time and magnetisation pattern of the drive. This is not surprising as hard drive recording media are designed to be magnetically stable and robust against thermal fluctuations.


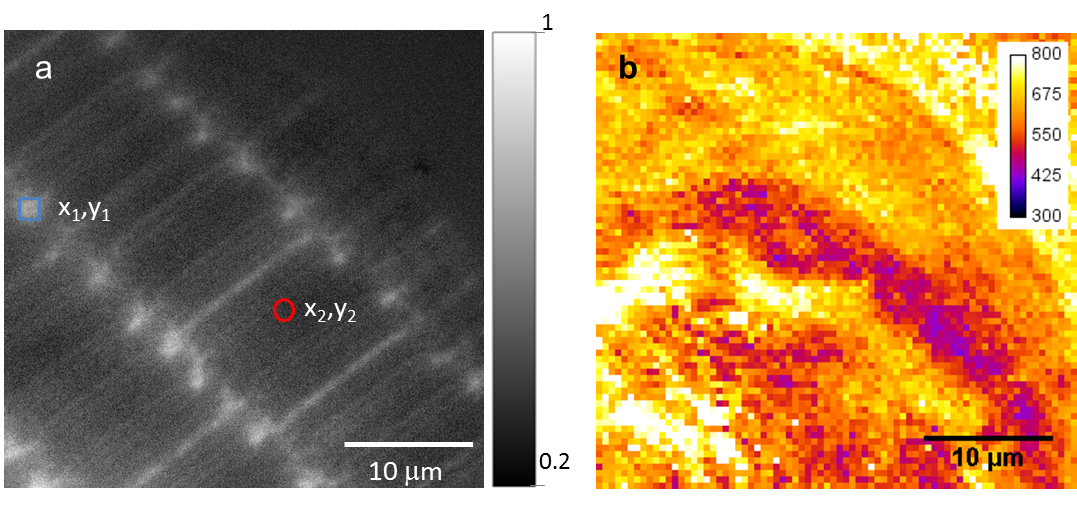


Supplementary Figure 5: a. ODMR based-magnetic image (B=0.9mT) of the commercial drive. b Corresponding spin relaxation time (*T_1_*) image of the same area of the drive with the calibration bar representing the *T_1_* time in µs.

The average *T_1_* time measured across the imaging chip was 630 ± 95 µs. The standard deviation of ~15% would result in a fluorescence intensity difference of 0.5%. The plot in Figure 4b shows the spin relaxation contrast observed between areas of high and low off-axis magnetic field is considerably larger (~4%), which verifies the dominant contribution to the contrast seen in Figure 4d arises from the spin relaxation contrast.

**References**

1.S. Steinert, F. Ziem, L. Hall, A. Zappe, et al., Nat Commun **4**, 1607 (2013).
